# Genetic polymorphisms and non-small-cell lung cancer: future paradigms

**DOI:** 10.1590/S1679-45082014RB2906

**Published:** 2014

**Authors:** Ramon Andrade Bezerra de Mello

**Affiliations:** 1 Serviço de Oncologia Médica, Instituto Português de Oncologia Francisco Gentil, Porto, Portugal;; 2 Departamento de Ciências Biomédicas e Medicina, Universidade do Algarve, Faro, Portugal.

**Keywords:** Lung neoplasms, Polymorphism, genetic, Tumor makers, biological, Prognosis

## Abstract

This article addresses some current issues about genetic polymorphisms studied in the non-small-cell lung cancer translational field. Furthermore, it discusses about new potential biomarkers regarding lung cancer risk and prognosis.

## INTRODUCTION

Currently, non-small cell lung cancer (NSCLC) is a neoplasm of worldwide importance due to its high incidence and mortality.^(1) ^Patients in stage IV according to the TNM Classification of Malignant Tumors, of the American Joint Cancer Committee (AJCC),^(2) ^have a median overall survival (OS) of 10 to 11 months after the treatments.^(3) ^In 2011, the group from the University of Porto and University of Minho, in Portugal, published a review^(3)^ that emphasized various studies related to the treatment of the advanced disease using treatments directed towards the main molecular pathways responsible for the carcinogenesis of NSCLC.^(4)^ The epidermal growth factor (EGF) and its receptor, EGFR, have been shown to be very significant pathways in the carcinogenesis of lung neoplasm. The mutation of the EGFR at exons 19 and 21 was recently described as a predictor of a good response to the therapeutic use of tyrosine kinase inhibitors as a first, second, or third line of systemic treatment for advanced NSCLC, especially adenocarcinomas in non-smokers and Asians.^(5-9)^ Additionally, the vascular endothelial growth factor (VEGF) is also intimately related to carcinogenesis of NSCLC.^(3)^ Some studies^(10-12) ^showed that certain genetic polymorphisms responsible for the regulation of the VEGF pathway may be associated with risk and prognosis in patients with NSCLC.^(13)^ In this way, the objective of this article was to briefly review important aspects related to the main target-therapies approved for treating advanced NSCLC and potential biomarkers.

## TARGET THERAPIES

### Vascular endothelial growth factor

In 2009, the AVAiL study was very important for the approval, in Europe and the United States, of the use of bevacizumab, a monoclonal antibody against VEGF, in advanced-stage NSCLC.^(14) ^Nevertheless, posterior analysis of these results implied discontinuation of bevacizumab in advanced NSCLC since the cost-benefit association was not confirmed as to an increase in OS (only about 2 months), according to the European Medicines Agency (EMA).^(15)^

### Epidermal growth factor

In 2010, other studies^(4) ^demonstrated the superiority of geftinib, an EGFR tyrosine kinase inhibitor as first line treatment of patients with advanced NSCLC, presenting with EGFR mutation at exons 19 and 21. Since then, there has been a great advance in the treatment results of these patients. The EGFR tyrosine kinase inhibitors showed excellent results as to longer OS in stage-IV patients, mutated EGFR, and especially Asian non-smokers.^(4)^

## EML4-ALK Fusion

The fusion of EML4-ALK (echinoderm microtubule-associated protein-like 4 – anaplastic lymphoma kinase) was identified as having a significant role in the clinical management of a small subgroup of patients (about 6%) with NSCLC, especially adenocarcinomas, who were non-smokers and young.^(16-19) ^Some studies in this regard,^(16,17,20-23)^ although yet with small number of patients, demonstrated that crizotinib, an ALK tyrosine kinase inhibitor, showed an excellent overall response (approximately 57%) to treatment in EML4-ALK-positive patients with advanced disease.^(16,17,20-23)^ Thus, the role of EML4-ALK and of crizotinib seems to be promising within this context, although broader Phase II studies are necessary to establish, with detail, all these aspects.

## BIOMARKERS AND GENETIC POLYMORPHISMS

There is, however, a great need to try to stratify patients using biomarkers related specifically to the risk of developing the neoplasm and to the prognosis in various stages. Nevertheless, a few practical questions also arise: to what extent is it possible to obtain biological samples for biomarker investigation? Are there plausible instruments for intervention in this stratified population? Can these approaches, in fact, influence the clinical progression of these patients? Currently, by the recommendations of the National Cancer Comprehensive Network (NCCN), in the United States, approve the use for low-dose computed axial tomography (CAT) of the chest to trace lung carcinoma in individuals aged over 55 years and a smoking load of more than 30 pack-years (PYs).^(24)^ In 2012, in Portugal, our group was able to demonstrate an association between the susceptibility of the NSCLC and genetic polymorphism of EGF+61 A/G.^(2^ ^5^^)^ Considering that the determination of this genetic polymorphism consists of a low-cost and practical test, since it is obtained from peripheral blood samples, we believe that the use of this biomarker may be useful in the future for improving tracking of lung carcinoma, optimizing the selection of patients elegible for low-dose chest CAT, and consequently, the financial resources available for this imaging test. On the other hand, we also know that the evaluation of germinating line genetic polymorphisms is performed by DNA extraction of peripheral blood using complete blood count (CBC) tubes containing EDTA (ethylenediaminetetraacetic acid). Thus, the implementation of certain genetic polymorphisms in clinical practice is extremely practical and feasible, with the potential to generate great benefit for the patients considered.^(26,27) ^Keeping these aspects in mind, a strong new trend in studies arises, which tries to investigate the role of genetic polymorphisms as prognostic biomarkers for NSCLC. Those that stand out are the pathways linking VEGF and the CLPTM1L (cleft lip palate transmembrane-like receptor 1).^(3,28) ^By means of genome wide association studies (GWAS,) polymorphic variants of susceptibility to lung cancer were detected in chromosomes 5p15.33, 6p21, and 15q25.^(28) ^Specifically, the variants rs2736100, located at intron 2 of telomerase reverse transcriptase (TERT) and rs401681 of CLPTM1L C/T, in 5p15.33, are associated with lung adenocarcinomas.

Therefore, from this point on, genetic polymorphisms have become an emphasized theme in translational studies, with the objective of creating a bridge between basic research and clinical applicability for the study of NSCLC in several European and worldwide centers.

## CONCLUSION

Finally, the clinical and therapeutic approaches of lung cancer have had great advances over the last few decades by means of translational studies that identified predictive biomarkers already established in clinical practice, such as the of EGFR mutation and the of ALK-EML4 fusion. These are associated with the choice of target therapies, such as erlotinib, gefitinib and, more recently, crizotinib. In the future, new biomarkers should appear with the use of certain polymorphic variations of the germinative line, such as EGF+61 A/G,VEGF – 460 C/T, and CLPTM1L C/T, as biomarkers of risk and prognosis of non-small cell lung cancer, and may be an additional help to clinical oncologists during treatment of these patients. Nevertheless, prospective and randomized studies are still necessary to validate the potential value of these findings.

## References

[1] Siegel R, Naishadham D, Jemal A (2012). Cancer statistics, 2012. CA Cancer J Clin.

[2] Goldstraw P, Crowley J, Chansky K, Giroux DJ, Groome PA, Rami-Porta R, Postmus PE, Rusch V, Sobin L (2007). International Association for the Study of Lung Cancer International Staging Committee; Participating Institutions. The IASLC Lung Cancer Staging Project: proposals for the revision of the TNM stage groupings in the forthcoming (seventh) edition of the TNM Classification of malignant tumours. J Thorac Oncol.

[3] de Mello RA, Costa BM, Reis RM, Hespanhol V (2012). Insights into angiogenesis in non-small cell lung cancer: molecular mechanisms, polymorphic genes, and targeted therapies. Recent Pat Anticancer Drug Discov.

[4] de Mello RA, Marques DS, Medeiros R, Araújo AM (2011). Epidermal growth factor receptor and K-Ras in non-small cell lung cancer-molecular pathways involved and targeted therapies. World J Clin Oncol.

[5] Shepherd FA, Rodrigues Pereira J, Ciuleanu T, Tan EH, Hirsh V, Thongprasert S, Campos D, Maoleekoonpiroj S, Smylie M, Martins R, van Kooten M, Dediu M, Findlay B, Tu D, Johnston D, Bezjak A, Clark G, Santabárbara P, Seymour L (2005). National Cancer Institute of Canada Clinical Trials Group. Erlotinib in previously treated non-small-cell lung cancer. N Engl J Med.

[6] Tanaka T, Matsuoka M, Sutani A, Gemma A, Maemondo M, Inoue A (2010). Frequency of and variables associated with the EGFR mutation and its subtypes. Int J Cancer.

[7] Rosell R, Cuello M, Cecere F, Santarpia M, Reguart N, Felip E (2006). Usefulness of predictive tests for cancer treatment. Bull Cancer.

[8] Kosaka T, Yatabe Y, Endoh H, Kuwano H, Takahashi T, Mitsudomi T (2004). Mutations of the epidermal growth factor receptor gene in lung cancer: biological and clinical implications. Cancer Res.

[9] Kosaka T, Yatabe Y, Onozato R, Kuwano H, Mitsudomi T (2009). Prognostic implication of EGFR, KRAS, and TP53 gene mutations in a large cohort of Japanese patients with surgically treated lung adenocarcinoma. J Thorac Oncol.

[10] Naik NA, Bhat IA, Afroze D, Rasool R, Mir H, Andrabi SI (2012). Vascular endothelial growth factor A gene (VEGFA) polymorphisms and expression of VEGFA gene in lung cancer patients of Kashmir Valley (India). Tumour Biol.

[11] Heist RS, Zhai R, Liu G, Zhou W, Lin X, Su L (2008). VEGF polymorphisms and survival in early-stage non-small-cell lung cancer. J Clin Oncol.

[12] de Mello RA, Ferreira M, Soares-Pires F, Costa S, Cunha J, Oliveira P (2013). The impact of polymorphic variations in the 5p15, 6p12, 6p21 and 15q25 Loci on the risk and prognosis of portuguese patients with non-small cell lung cancer. PLoS One.

[13] de Mello RA, Luis M, Araújo A, Reis RM, Hespanhol V, Mehta JL, Dhalla NS (2013). The role of genetic polymorphisms in the angiogenesis pathway and non-small cell lung cancer tumor behavior: implications in risk assessment and clinical outcome. Biochemical Basis and Therapeutic Implications of Angiogenesis.

[14] Reck M, von Pawel J, Zatloukal P, Ramlau R, Gorbounova V, Hirsh V (2009). Phase III trial of cisplatin plus gemcitabine with either placebo or bevacizumab as first-line therapy for nonsquamous non-small-cell lung cancer: AVAil. J Clin Oncol.

[15] Reck M, von Pawel J, Zatloukal P, Ramlau R, Gorbounova V, Hirsh V, Leighl N, Mezger J, Archer V, Moore N, Manegold C (2010). BO17704 Study Group. Overall survival with cisplatin-gemcitabine and bevacizumab or placebo as first-line therapy for nonsquamous non-small-cell lung cancer: results from a randomised phase III trial (AVAiL). Ann Oncol.

[16] Soda M, Choi YL, Enomoto M, Takada S, Yamashita Y, Ishikawa S (2007). Identification of the transforming EML4-ALK fusion gene in non-small-cell lung cancer. Nature.

[17] Choi YL, Soda M, Yamashita Y, Ueno T, Takashima J, Nakajima T, Yatabe Y, Takeuchi K, Hamada T, Haruta H, Ishikawa Y, Kimura H, Mitsudomi T, Tanio Y, Mano H (2010). ALK Lung Cancer Study Group. EML4-ALK mutations in lung cancer that confer resistance to ALK inhibitors. N Engl J Med.

[18] Araújo A, Coelho A, de Mello RA, Azevedo I, Soares M, Queiroga H (2012). Personalizing medicine: strategies for implementing the evaluation of anaplastic lymphoma kinase rearrangement in non-small-cell lung cancer in Portugal. Rev Port Pneumol.

[19] De Mello RA, Araújo A (2013). Anaplastic lymphoma kinase gene rearrangement and non-small cell lung cancer management: a step forward in personalized therapy. Clinics.

[20] Camidge DR, Bang YJ, Kwak EL, Iafrate AJ, Varella-Garcia M, Fox SB (2012). Activity and safety of crizotinib in patients with ALK-positive non-small-cell lung cancer: updated results from a phase 1 study. Lancet Oncol.

[21] Kwak EL, Bang YJ, Camidge DR, Shaw AT, Solomon B, Maki RG (2010). Anaplastic lymphoma kinase inhibition in non-small-cell lung cancer. N Engl J Med.

[22] Shaw AT, Yeap BY, Mino-Kenudson M, Digumarthy SR, Costa DB, Heist RS (2009). Clinical features and outcome of patients with non-small-cell lung cancer who harbor EML4-ALK. J Clin Oncol.

[23] Shaw AT, Yeap BY, Solomon BJ, Riely GJ, Gainor J, Engelman JA (2011). Effect of crizotinib on overall survival in patients with advanced non-small-cell lung cancer harbouring ALK gene rearrangement: a retrospective analysis. Lancet Oncol.

[24] Team National Lung Screening Trial Research, Church TR, Black WC, Aberle DR, Berg CD, Clingan KL, Duan F, Fagerstrom RM, Gareen IF, Gierada DS, Jones GC, Mahon I, Marcus PM, Sicks JD, Jain A, Baum S (2013). Results of initial low-dose computed tomographic screening for lung cancer. N Engl J Med.

[25] de Mello RA, Ferreira M, Costa S, Costa BM, Pires FS, Neves I (2012). Association between EGF +61 genetic polymorphisms and non-small cell lung cancer increased risk in a Portuguese population: a case-control study. Tumour Biol.

[26] Hu-Lieskovan S, Vallbohmer D, Zhang W, Yang D, Pohl A, Labonte MJ (2011). EGF61 polymorphism predicts complete pathologic response to cetuximab-based chemoradiation independent of KRAS status in locally advanced rectal cancer patients. Clin Cancer Res.

[27] De Mello RA (2012). Back to EGF+61 genetic polymorphisms and lung cancer risk: looking to the future!. Sao Paulo Med J.

[28] Landi MT, Chatterjee N, Yu K, Goldin LR, Goldstein AM, Rotunno M (2009). A genome-wide association study of lung cancer identifies a region of chromosome 5p15 associated with risk for adenocarcinoma. Am J Hum Genet.

