# Supplementary material for: Genetic polymorphisms and non-small-cell lung cancer: future paradigms
Source: Einstein (Sao Paulo). 2014 Oct-Dec;12(4):524–6. doi: 10.1590/S1679-45082014RB2906 (PMC4879925; doi:10.1590/S1679-45082014RB2906)
Supplement: Translation

# Translation: Polimorfismos genéticos e carcinoma de pulmão de células não pequenas: os paradigmas do futuro

**Authors:** Ramon Andrade Bezerra de Mello

**Affiliations:** 1 Serviço de Oncologia Médica, Instituto Português de Oncologia Francisco Gentil, Porto, Portugal;; 2 Departamento de Ciências Biomédicas e Medicina, Universidade do Algarve, Faro, Portugal.

**Keywords:** Neoplasias do pulmão, Polimorfismo genético, Marcadores biológicos de tumor, Prognóstico

## Abstract

O presente artigo faz uma abordagem de questões atuais sobre os polimorfismos genéticos, que têm sido objeto de estudo translacional no contexto do carcinoma de pulmão de células não pequenas. Além disso, discute os novos potenciais biomarcadores de risco e prognóstico.

## INTRODUÇÃO

Atualmente, o carcinoma de pulmão de células não pequenas (CPCNP) consiste numa neoplasia de importância mundial, devido à sua elevada incidência e mortalidade.^(1)^ Doentes com estádio IV, de acordo com a Classificação de Tumores Malignos (Classificação TNM), do *American Joint Cancer Comittee* (AJCC),^(2)^ apresentam uma sobrevivência global (SG) mediana de 10 a 11 meses após os tratamentos.^(3)^ Em 2011, o grupo da Universidade do Porto e da Universidade do Minho, em Portugal, publicou revisão^(3)^ que enfatizou os vários estudos relacionados ao tratamento da doença avançada utilizando terapêuticas dirigidas às principais vias moleculares responsáveis pela carcinogênese do CPCNP.^(4)^ O fator de crescimento epidérmico (EGF, do inglês *epidermal growth factor*) e seu receptor (EGFR) têm sido demonstrados como vias muito importantes na carcinogênese da neoplasia do pulmão. A mutação do EGFR nos éxons 19 e 21 foi recentemente descrita como preditora de boa resposta ao uso de terapêuticas inibidoras da tirosina quinase em primeira, segunda ou terceira linha de tratamento sistêmico para CPCNP avançado, especialmente os adenocarcinomas, não fumantes e asiáticos.^(5-9)^ Além disso, o factor de crecimento endotelial vascular (VEGF, *do inglês vascular endothelial growth factor*) também encontra-se intimamente relacionado com a carcinogênese do CPCNP.^(3)^ Alguns estudos^(10-12)^ mostraram que determinados polimorfismos genéticos responsáveis pela regulação da via do VEGF podem estar associados ao risco e ao prognóstico em doentes com CPCNP.^(13)^ Dessa forma, o objetivo deste artigo foi rever brevemente aspectos importantes relacionados às principais terapêuticas-alvo aprovadas para o tratamento do CPCNP avançado e potenciais biomarcadores.

## TERAPÊUTICAS-ALVO

### Fator de crescimento endotelial vascular

Em 2009, o estudo AVAiL foi bastante importante para a aprovação, na Europa e nos Estados Unidos, da utilização do bevacizumabe, um anti-corpo monoclonal contra o VEGF, no CPCNP em estádio avançado.^(14)^ No entanto, a análise posterior desses resultados implicou na retirada da utilização do bevacizumabe no CPCNP avançado, pois, segundo a *European Medicines Agency* (EMA), não se comprovou a relação custo-benefício, no que diz respeito ao aumento da SG (cerca de apenas 2 meses).^(15)^

### Fator de crescimento epidérmico

Em 2010, outros estudos^(4) ^demonstraram a superioridade do geftinib, um inibidor da tirosina quinase do EGFR, no tratamento de primeira linha de pacientes com CPCNP avançado, apresentando a mutação do EGFR nos éxons 19 e 21. A partir de então, houve grande avanço nos resultados do tratamento desses pacientes. Os fármacos inibidores da tirosina quinase do EGFR mostraram excelentes resultados no que diz respeito ao aumento da SG nos doentes em estádio IV, EGFR mutado e principalmente não fumantes asiáticos^(4)^.

## FUSÃO EML4-ALK

A fusão do EML4-ALK (sigla em inglês para* echinoderm microtubule-associated protein-like 4 – anaplastic lymphoma kinase*) foi identificada como tendo um importante papel na abordagem clínica de um pequeno subgrupo de pacientes (cerca de 6%) com CPCNP, especialmente adenocarcinomas, não fumantes e jovens.^(16-19)^ Nesse contexto, alguns estudos,^(16,17,20-23)^ embora ainda com poucos pacientes, demostraram que o crizotinib, uma molécula inibidora da tirosina quinase do ALK, apresentou excelente resposta global (aproximadamente 57%) ao tratamento em pacientes com doença avançada EML4-ALK positivos.^(16,17,20-23)^ Assim, o papel do EML4-ALK e do crizotinib parece promissor nesse contexto, embora estudos mais alargados, de fase II, são necessários para estabelecer, com pormenores, todos esses aspectos.

## BIOMARCADORES E POLIMORFISMOS GENÉTICOS

Há, portanto, uma imensa necessidade de tentar estratificar os pacientes utilizando-se biomarcadores relacionados especificamente ao risco de desenvolver a neoplasia e ao prognóstico em diversos estádios. No entanto, surgem também algumas perguntas práticas: até que ponto é possível obter amostras biológicas para a pesquisa de biomarcadores? Existem instrumentos plausíveis para intervir nessa população estratificada? Será que essas abordagens podem influenciar, de fato, a evolução clínica desses pacientes? Atualmente, foi aprovada, pelas recomendações americanas do *National Cancer Comprehensive Network* (NCCN), a utilização de tomografia-axial-computadorizada (TAC) torácica de baixa dose para o rastreio do carcinoma de pulmão em indivíduos com mais de 55 anos e carga tabágica acima de 30 Unidades Maço Ano (UMA).^(24)^ Em 2012, em Portugal, o nosso grupo conseguiu demonstrar uma associação entre a suscetibilidade do CPCNP e o polimorfismo genético do EGF+61 A/G.^(25)^ Considerando que a determinação desse polimorfismo genético consiste num exame de baixo custo e bastante prático, por ser obtido de amostras de sangue periférico, acreditamos que a utilização desse biomarcador pode, no futuro, ser útil para melhorar o rastreio do carcinoma de pulmão, otimizando a seleção de pacientes elegíveis para TAC de baixa dose e, consequentemente, os recursos financeiros disponíveis para esse exame de imagem. Por outro lado, também sabemos que a avaliação de polimorfismos genéticos da linha germinativa são efetuados por meio da extração de DNA por sangue periférico, utilizando tubos de hemograma, contendo EDTA (do inglês *ethylenediaminetetraacetic acid*). Assim, a implementação de determinados polimorfismos genéticos na prática clínica é algo extremamente prático e factível, com o potencial de gerar grande benefício para os pacientes em questão.^(26,27)^ Tendo em vista esses aspectos, nasce uma forte vertente de estudos, que tentam investigar o papel de polimorfismos genéticos como biomarcadores de prognóstico do CPCNP. Em destaque, estão as vias do ligando VEGF e do receptor do CLPTM1L (do inglês *cleft lip palate transmembrane-like receptor 1*).^(3,28)^ Por meio de estudos de associação de genoma completo (GWAS, *genome wide association studies*) foram detectadas variantes polimórficas de suscetibilidade ao câncer de pulmão nos cromossomo 5p15.33, 6p21 e 15q25.^(28)^ Especificamente, as variantes rs2736100, localizadas no íntron 2 do TERT *(telomerase reverse transcriptase*) e rs401681 do (CLPTM1L C/T), em 5p15.33, estão associadas a adenocarcinomas do pulmão.

Portanto, a partir desse ponto, os polimorfismos genéticos passaram a ser um tema de destaque em estudos translacionais, com o objetivo de criar uma ponte entre a pesquisa básica e a aplicabilidade clínica para o estudo do CPCNP em vários centros europeus e ao nível mundial.

## CONCLUSÃO

Em resumo, as abordagens clínica e terapêutica do carcinoma de pulmão tiveram um grande avanço nas últimas décadas, por meio de estudos translacionais que identificaram biomarcadores preditivos já estabelecidos na prática clínica, como a mutação do EGFR e a fusão ALK-EML4, que se econtram associados à escolha de terapêuticas-alvo como o erlotinibe, gefitinibe e, mais recentemente, o crizotinib. No futuro, novos biomarcadores devem surgir, por meio da utilização de determinadas variações polimórficas da linha germinativa, como o EGF+61 A/G, o VEGF – 460 C/T e o CLPTM1L C/T, como biomarcadores de risco e prognóstico do carcinoma de pulmão de células não pequenas, podendo constituir uma ajuda adicional aos oncologistas clínicos durante a abordagem desses pacientes. No entanto, estudos prospectivos e randomizados são ainda necessários para validar o potencial valor desses achados.
